# Isolated cystic tuberculosis of scapula; case report and review of literature

**DOI:** 10.1186/1749-799X-5-72

**Published:** 2010-10-08

**Authors:** Sujit K Tripathy, Ramesh K Sen, Anurag Sharma, Tajir Tamuk

**Affiliations:** 1 Department of Orthopaedics, Postgraduate Institute of Medical Education and Research, Chandigarh, India

## Abstract

Tubercular osteomylitis of scapula is extremely rare. The isolated involvement of this flat bone without any primary focus confuses the surgeon with other pathology and as a result there is always delay in diagnosis. This article discusses about an isolated multicystic tubercular lesion of scapula which remained untreated for about two years as the primary physician biased with the history of trauma and suspected it to be a post-traumatic hematoma. MRI picture was deceptive. Finally, diagnosis was established by fine needle aspiration which showed typical epitheloid granuloma on histology. Lack of awareness and nonspecific radiological picture may cause delay in diagnosis of scapular tuberculosis. Tuberculosis is an important consideration in isolated scapular swelling particularly in endemic regions and the histological diagnosis by fine needle aspiration may be helpful in cases of doubtful radiological pictures.

## Background

Resurgence of tuberculosis with the rising burden of acquired immunodeficiency syndrome has created a major problem before health professionals [1]. Their atypical presentations in unusual sites lead to delay in diagnosis or misdiagnosis [2-9]. Tuberculosis of scapula is an extremely rare presentation of osteoarticular tuberculosis and only nine cases of their isolated involvement have been reported till date [3-11]. We report a case of multicystic tubercular lesion of scapula in a young active male. The primary involvement this flat bone without any other focus makes this article unique. The diagnostic dilemma and treatment has been described in brief.

## Case Description

**Figure 1:**
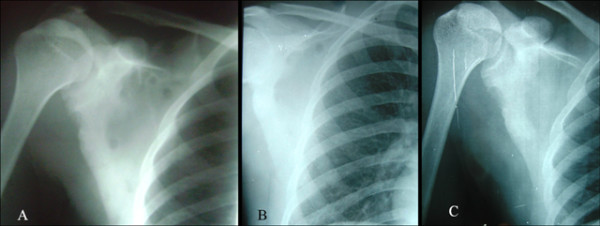
**A. Initial radiograph of right scapula (at the time of presentation) showing multiple cystic lesions over the scapular body with surrounding sclerosis B**. After 6 months of anti tubercular therapy, most of the cystic lesions healed. Still one cystic cavity is noticed on supero-medial aspect C. After 2 years, the cystic lesions have completely healed.

**Figure 2:**
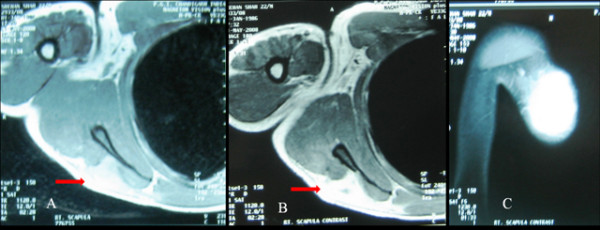
**A, B, C: MRI scan in axial and coronal cut sections showing hyperintense image on T1W and T2W sequence, but no significant enhancement noticed in postcontrast images**.

A 22 year male presented with progressively increasing pain and swelling in the right upper back since 2 years. He had history of fall from a height of about 6 feet before two years. There were no injuries other than superficial skin abrasions over the site. After which he developed the pain and swelling in the above region for which he was treated with analgesic and local anti-inflammatory medication by the local physician. The symptoms subsided to some extent but did not relieved completely. He consulted many physicians but to receive the same treatment. The patient ignored the symptoms and continued to manage his daily activities with analgesics on demand. After 20 months he had significantly diminished pain but to have a massive swelling in that region. When he presented to us, the swelling appeared to be arising from right scapula that was mild tender with minimal rise in temperature. The size of the mass was 15×10 cms with a globular shape. It was non-pulsatile with soft to firm consistency. There was no lymphadenopathy or hepato-splenomegaly. Radiograph revealed multiple cystic lesions in the right scapular body with sclerotic margin and overlying soft-tissue involvement [Fig 1A]. The glenohumeral joint did not show any evidence of involvement. Other than a raised ESR (ESR = 74 mm/hr), rest of the haematological parameters were with in normal range. MRI of the lesion was advised with clinical suspicion of malignancy. It showed altered signal in the subcutaneous plane with hyperintense T1W and T2W images. No signal alterations and enhancement were noticed on fat saturated images and post-contrast images. It was dissecting into the fibers of infraspinatus muscle on the dorsal aspect of scapula [Fig 2A, B, C]. The scapular cortex was found to be discontinuous at that level. The likely possibility of hematoma was put forward by the radiologist.

**Figure 3:**
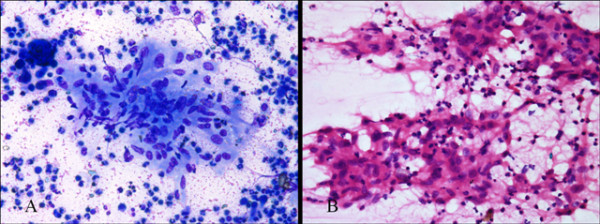
**A, B: Leishman and H&E staining of aspirate showing typical epitheloid granuloma with inflammatory cells and proliferating blood vessels**.

Fine needle aspiration of the mass reveled a creamy aspirate which was stained for histopathological evaluation as well as sent for culture and sensitivity and staining for bacteria and fungus. The histological finding showed typical epitheloid granuloma in a background of marked inflammation comprising of sheets of neutrophils, histiocytes, plasma cells, and few reactive lymphocytes [Fig 3A, B]. It was consistent with tuberculosis. However the organism could not be visualized in acid fast stain. Chest x-ray, urine and sputum examination was normal. Monteux test showed induration of 20× 20 mm. HIV ELISA was found to be negative. Culture of the aspirate in Lowenstein medium showed the growth of the tubercle bacilli. Based on the histological findings, the patient was treated with antitubercular therapy for 12 months. There was complete resolution of the lesion both clinically and radiographically at the end of 2 years [Fig 1B, C].

## Discussion

Osteoarticular tuberculosis constitutes only 1-2% of all tuberculosis [10]. Though spine is considered as the most common site of involvement in skeletal TB followed by femur, tibia and small bones of hand; virtually no bone is immune to the bacilli. Flat bone like Scapula is a rare site for bony tuberculosis. Literature till date has only 17 cases, of which 9 are of isolated involvement [1-16][Table 1]. Bone TB result from hematogenous or lymphatic dissemination of the bacilli from a primary focus of lungs, lymph node or gut. Isolated bone involvement without any primary focus and without history of TB contact in a well active young patient raises question about its mode of spread to this unusual site like scapula. Direct inoculation of the bacilli to the muscle through needle while giving injection and during trauma is a well known fact [17]. Scapula being a superficial bone on the dorsal aspect can be easily penetrated by any sharp objects. The definite history of fall as in the present case led to abrasion and contusion over the upper back region in the right side. The organism might have inoculated during this fall and have induced osteomylitis. Because of the habit of spitting, coughing and sneezing in open air, most of the pulmonary TB patient spread the disease to the environment and hence the soil, sand and dust in endemic areas are studded with plenty of bacilli.

**Table 1:** Scapular tuberculosis as available in literature till date

	Study	No. of patients	Age/sex	Area of Scapula involved	Presenting complaints	Other focus	Treatment
1	Lafond 1958 [13]	One	NA	NA	NA	NA	NA

2	Martini et al. 1986 [2]	One	NA	Acromian	NA	NA	NA

3	Shannon et al. 1990 [14]	One	4/M	Scapula	Pain and swelling in left shoulder	Multifocal cystic lesion, with Right ileum involvement	ATT

4	Mohan et al. 1991 [3]	One	23/F	Body of scapula	Pain and swelling	Isolated	Drainage and ATT

5	Gusati et al. 1997 [4]	One	NA	Spine of scapula	Pain	Isolated	Surgery and ATT

6	Vohra et al. 1997 [5]	One	NA	Body of scapula	NA	Isolated	NA

7	Kam et al. 2000 [6]	Two	31/M	Acromian,	1) Pain and swelling	Isolated	Debridement and curettage + ATT
			
			22/F	Lareral border of scapula	2) Incidental finding	Multifocal (T12 and L2 vertebrae; upper part of the Rt sacroiliac Joint)	ATT

8	Greenhow and Weintrub 2004 [15]	One	14/F	Inferior aspect of the left scapula	Enlarging, nontender mass	Cystic lesion with a soft tissue component, located dorsal to the Lt scapula	Scapular mass excision

9	Stones and Schoeman 2004 [16]	One	42/M	Scapula	Discharging sinus	Multifocal tuberculosis involving maxilla, parital bones and spine	Died

10	Husen et al. 2006 [7]	One	18/M	Spine of scapula near neck	Diffuse pain	Isolated	ATT

11	Srivastav et al 2006 [8]	One	26/F	Inferior angle of scapula	Pain and swelling	Isolated	ATT

12	Solav S 2007 [11]	Three	54/F	Medial margin and spine of scapula	Pain	Isolated	ATT
			
			26/M	Rt scapula	Occiptal headache and backpain (incidental finding on bone scan)	Multifocal (sternum, rib, vertebra)	NA
			
			40/M	Rt scapula	Rt shoulder pain and backache	Multifocal (L4 vertebra)	NA

13	Jain et al 2009 [9]	One	14/M	Body of scapula involving glenoid margin	Rt Pain swelling and discharging sinus	Isolated	ATT

14	Singh et al 2009 [10]	One	49/F	Inferior angle of Lt scapula	Pain and swelling	Isolated	ATT

M: Male, F: Female, Rt: Right, Lt: Left, NA: not available, ATT: Antitubercular therapy

The indolent nature of the disease and lack of constitutional symptoms often causes late presentation. Raised ESR and positive Monteux test are though consistent findings; these are not diagnostic of tuberculosis in endemic areas. Radiographic findings in tubercular osteomylitis include radiolucent lesion with irregular margin and surrounding sclerosis [6,7,9,10]. The cystic cavitary lesions on radiograph are highly nonspecific and simulate with pyogenic osteomylitis, fungal infection, metastasis, telengiectactic osteosarcoma, aneurysmal cyst, sarcoidosis, eosinophilic granuloma or chordoma [6,10,11]. Differentiation of TB from all these differentials may not be possible without tissue biopsy. MRI scan may be sometime deceptive. The present study did not show any enhancement after postcontrast evaluation and the radiologist put the possibility of hematoma dissected into the infraspinatous muscle. Morris reported that confirmation of musculoskeletal tuberculosis is solely based on identification of epitheloid granuloma and caseous necrosis or tubercle bacilli in fine needle aspirates or on tissue culture studies [12]. Masood reported that FNAC is a good alternative to open biopsy as it can show the granulomatous reaction in 73% of time, bacteria in 64% and positive culture in 83% of time [18]. Accordingly the present case was diagnosed on the basis of histological findings which revealed epitheloid granuloma on histology. The culture report further supported the diagnosis.

Many authors feel that in the absence of giant sequestra, most of the tubercular osteomylitis can be treated with antitubercular therapy only. The effective multidrug chemotherapy can resolve the sequestra and can cause early disease remission [10]. Twelve months of antitubercular therapy in the present case had completely healed the lesion.

## Conclusion

Tubercular osteomylitis is an important cause of isolated scapular swelling in endemic areas. Lack of awareness and absence of constitutional symptoms, nonspecific radiographic findings and antecedent history of trauma may bias the surgeons and physician. Histology remains as the ultimate diagnostic tool. The bacilli may not be isolated at all time and treatment has to be started on the basis of granuloma. With the advent of highly effective multidrug chemotherapy, most of these can be successfully treated with antitubercular therapy alone.

## Abbreviations

MRI: Magnetic resonance imaging; ESR: Erythrocyte Sedimentation Rate; TB: Tuberculosis; HIV ELISA: Human Immunodeficiency Virus Enzyme Linked Immunosorbent Assay; ATT: Antitubercular therapy; FNAC: Fine Needle Aspiration Cytology;

## Consent

"Written informed consent was obtained from the patient for publication of this case report and any accompanying images. A copy of the written consent is available for review by the Editor-in-Chief of this journal."

## Competing interests

The authors received no financial or other type of support to carry out this study; there is no conflict of interests. This is an original article and has not been published in any other journal.

## Authors' contributions

SKT and RKS managed the patient. SKT and AS prepared the manuscript. TT assisted in review of literature and revising the manuscript. RKS revised the manuscript and provided intellectual content. All authors have read and approved the final manuscript.
